# Dualities of dementia illness narratives and their role in a narrative economy

**DOI:** 10.1111/1467-9566.12729

**Published:** 2018-04-16

**Authors:** Alexandra Hillman, Ian Rees Jones, Catherine Quinn, Sharon M. Nelis, Linda Clare

**Affiliations:** ^1^ WISERD: Wales Institute of Social & Economic Research Data & Methods, School of Social Sciences Cardiff University UK; ^2^ Centre for Research in Ageing and Cognitive Health (REACH) School of Psychology University of Exeter UK; ^3^ PenCLAHRC University of Exeter Medical School UK; ^4^ Wellcome Centre for Cultures and Environments of Health University of Exeter UK

**Keywords:** advocacy, Alzheimer's disease, carers, illness narratives, living well, narrative economies

## Abstract

The concept of ‘narrative economies’ has recently been proposed as a set of exchange relationships that, through biography and story‐telling, facilitate access to resources and act as a source of value. We utilise this concept to inform our analysis of 18 qualitative interviews with five people with dementia and four informal carers. Our participants are members of a pre‐existing group of dementia advocates, representing the voices of those living with the condition. There are a growing number of people in the early stages of dementia – like our participants – being called upon to account for their experience, as a means of developing a politicised ‘collective illness identity’. These interviews present an opportunity to study a group of people who are actively involved in speaking as, and for, people with dementia. Four themes emerged from the data: becoming a voice of or for people with dementia; biographical reinforcement; responsibilisation; and resistance. These themes illustrate the ways in which people with dementia participate in their own identity construction and, as representatives of those living with dementia, they also illustrate the ways in which illness narratives produce material and symbolic value.

## Introduction

Combatting the challenges posed by dementia has become a priority, across nations, for policymakers (WHO 2015) and for biomedical science and research communities (Winbald *et al*. 2016). Connected to this growing recognition has been an increased awareness of, and interest in, the condition in the wider public (Peel 2014, Van Gorp and Vercruysse 2012). These developments have created an appetite for stories about what it is like to live with dementia, not only from the perspective of those caring for people with dementia, but also, importantly, from people with dementia themselves. With medical and policy initiatives aligned to focusing on diagnosing those with dementia at an earlier stage (DOH 2016), it has become increasingly possible to hear the previously unheard voice of people with early signs of dementia (Beard 2004). We can see this manifested in many ways including depictions in mainstream press, in dementia research which increasingly includes people with dementia as co‐producers of research (‘nothing about us without us’ – Bryden 2015), books and collections of personal accounts describing what it is like to live with dementia (such as Jennings's 2014 *Welcome to our World*), and third sector campaigns in which the voice of those with dementia is a central focus (the UK Alzheimer's Society recent FADE campaign^1^ ). There has also been a proliferation of academic contributions that have sought to represent the voices of those living with dementia, particularly since the turn of the century (see for example Beard *et al*. 2009b, Moran 2001, Phinney 2002, Ryan *et al*. 2009, Sterin 2002, Taylor 2006). The foundations of this work began with Malcolm Goldsmith's (1996) book on hearing the voices of the people with dementia and was further developed by Tom Kitwood's work on personhood in dementia, ideas that were drawn together in his 1997 book. These dementia narratives play an important role in shaping the socio‐political landscape of dementia, contributing to a reframing of dementia as a ‘manageable disability’ (Beard *et al*. 2009b). These personal stories have the potential to challenge traditional media representations of dementia which tend to either reproduce panic and anxiety through ‘tales of tragedy’ (Basting 2009) that describe the condition as a living death (Behuniak 2011), or herald lifestyle changes or choices as being the key to avoiding or preventing dementia in the first place –a ‘panic‐blame’ framework (Peel 2014).

Our participants’ stories are conceptualised as dementia illness narratives and we explore these narratives through Burchardt's (2016) concept of narrative economies. Research on illness narratives has a historic and continuing influence on medical sociology's understanding of illness (Kleinman 1988) as well as what it means to live with different diseases (Ezzy 2000, Frank 1995, Hyden 1995). This work stresses the moral quality of illness narratives (Grey 2001) and the connections they reveal between individual experiences and deeper social and cultural meanings attached to illness and suffering (Bury 2001, Hinton and Levkoff 1999). Illness can pose a significant threat to a person's moral status due to its association with loss of control and competence in daily life, characteristics deeply ingrained in Western values (Becker 1997). Dementia is therefore particularly troubling, as the illness represents a gradual but progressive loss of precisely those characteristics. Dementia illness narratives provide the means through which the storyteller is able to present a form of coherence in the face of the disordering effects of the disease. These self‐narrations offer people the opportunity to interpret and appropriate their approach to living with the condition and thus to reassert their moral status. Illness narratives also help to construct a collective voice, accumulated through the telling of personal stories. Such commonality is significant in adding potency to individual stories and in recognising their implications for health and social care services, to the allocation of resources and to the wider recognition and understanding of dementia amongst communities.

We examine a set of interviews undertaken with people with dementia and their relatives who self‐identify as representatives of those who live with the condition. Their accounts are viewed as examples of the kinds of dementia illness narratives that are increasingly being co‐produced, circulated and repeated as a means of giving voice to this previously unheard community (see Bartlett and O'Connor 2010, Harris 2002, Hubbard *et al*. 2003, McKeown *et al*. 2010, Nazarko 2015). We situate these accounts in their wider context, highlighting the circulation of particular aspects of our participants’ stories that are shown to both reflect and contribute to a wider socio‐political landscape of dementia. In this sense, the interviews we present are both highly personal, reflecting the distinct ways in which dementia is experienced in the day to day lives of individuals, while also being socially and culturally framed by manifold meanings of dementia. When considered in tandem with the political nature of the way in which these stories come to represent the collective experience of dementia and the accompanying imperatives for social recognition and support, they relate to personal troubles and public issues (Mills 1959).

Marion Burchardt's (2016) work on narrative economies provides a conceptual framework for understanding the linkages and power dynamics between HIV positive people, activism and institutions in Africa; this framework provides an important bridge between institutions and narrative practices. Although Burchardt's substantive work focuses on HIV activism in Africa, the concept of narrative economies is useful for understanding the ways in which illness narratives play a significant role in shaping meanings of a condition at individual, organisational and societal levels. Our work explores the duality of the stories told by our participants, attending to the specific ways in which those living with dementia in the UK perform their identities while also recognising the ways in which these self‐narrations have the potential to ‘facilitate access to resources, knowledge and positions and thus acquire material and symbolic currency as they circulate’ (Burchardt 2016: 593). Burchardt's theorisation of narrative economies has four distinct parts: first, that autobiographical narratives produce surplus value in institutional contexts; second, that stories are implicated in exchange relationships in which their value is actualised; third, that the value of narrative is accrued in the process of story‐telling leading to ‘staged performances of authenticity’; and fourth, that the re‐telling of stories may produce biographical alienation (Reiter 2012) in which people become divorced from their own narratives (Burchardt 2016).

By focusing our attention specifically on those who self‐identify as representatives of the dementia community (both those with dementia and those living with and caring for those with dementia), we are able to gain insight into the kinds of dementia illness narratives of those seeking to act as representative voices, who contribute to the growing social movement of dementia activism. These interviews also provide examples of specific instances of storytelling and ‘performances of authenticity’ (Burchardt 2016). Of course these stories travel beyond the individuals who tell them and are able to penetrate institutions but this is not the focus of our article; instead, we explore the content of our participants’ narratives in the context of Burchardt's framework, to consider their potential implications for influencing the landscape of dementia.

To summarise, this article addresses three interconnected analytical aims:

1. To identify the kinds of representations of dementia present in the accounts of those who speak for people with dementia.
2. To situate these stories within their wider social and cultural contexts, to ascertain the extent to which they reflect, contribute to or challenge existing representations of dementia.
3. To utilise Burchardt's work (2016) to consider what the implications might be of their circulation and accumulation in a narrative economy of dementia.

## The study

### Method

The material presented in this article is drawn from 18 qualitative interviews, undertaken as part of the Improving the experience of dementia and enhancing active life (IDEAL) longitudinal cohort study of living well with dementia.

### Ethical approval

Ethical approval was obtained from the University Research Ethics Committee and the relevant NHS Local Research Ethics Committee. Written informed consent was given by all the study participants. In order to maintain confidentiality, all participants’ details were anonymised and pseudonyms were used in the transcripts of the interviews.

### Participants

The participants presented in this study are people living with dementia and their informal carers; their names and occupations have been changed for the purposes of anonymity. The participants were accessed from an existing group set up to engage in advocacy and represent the views of people living with dementia and the group were involved in speaking for people with dementia across a number of domains, including: working on community projects; advising on research; advising on care provision and policy initiatives; public speaking on the subject of living with dementia; writing about their experience; and taking part in media interviews. The study participants included four couples and one single person: Jack (59) who worked in education and is living with Alzheimer's disease and as his wife Beatrix (74); Fred (76) who worked in printing who is living with vascular dementia and his wife Ethel (75); Julia (79) who was both an accountant and a full‐time housewife and mother and is now living with Alzheimer's disease and her husband John (77); Oscar (83), who worked in finance and is living with mixed dementia and his wife Martha (71); and Jane (49) who was a carer and is living with vascular dementia and lives alone (see Table 1). Due to the opportunistic nature of the sample, resulting from accessing an existing advocacy group, we were unable to sample for specific types of dementia. However, our participants’ diagnoses reflected the most frequent causes of dementia, being diagnosed with either Alzheimer's disease or vascular dementia. The symptoms and challenges they described experiencing were similar, irrespective of their specific diagnosis. The participants living with dementia were all able to give consent to take part in the research, they had all been living with a diagnosis for at least three years at the time of their interviews and were in the early to middle stages of their dementia.

**Table 1:** : Study participants

Participant living with dementia	Diagnosis	Age	Participating relative	Age of relative
Jane	Vascular dementia	49	None	N/A
Jack	Young onset Alzheimer's disease	59	Beatrix (wife)	74
Fred	Vascular dementia	76	Ethel (wife)	75
Julie	Alzheimer's disease	79	John (husband)	77
Oscar	Mixed dementia	83	Martha (wife)	71

### Data collection

Participants were interviewed in their own homes between October 2015 and February 2016. The interviews followed a semi‐structured interview schedule and were audio‐recorded for later transcription. The participants were interviewed twice, a few months apart, in order to explore changes over time and how this impacted upon their ability to live well with dementia, both personally and in the context of people with dementia as a group. Part of the interest in interpreting the follow‐up interviews for the purposes of this article arose from the ways in which people's narratives and instances of story‐telling develop over time. In particular, through engaging with advocacy work, our participants might become more experienced in constructing and telling their stories.

Participants reflected on whether, and how, their lives had changed as a result of the dementia, on the aspects of their lives that enabled them to live well, and on the challenges they experienced in adapting to changes. These accounts could therefore be accurately described as illness narratives; they provide subjective accounts of the ways in which dementia is perceived as well as stories of adaptation to living with the condition by the person themselves and by others (Riessman 2003). Stories are always situated in their social and cultural contexts (Coffey and Atkinson 1996) through the culturally informed conventions and rules of story‐telling, including imagery and metaphor. Taking a narrative approach therefore attends to the ways in which stories can be reinforced, changed or passed on to affect other stories (Frank 1995). This is particularly helpful in seeking to understand the accounts of those who wish to represent what it is like to live with dementia – not just to reflect their personal experience but to use their story as a means of affecting others.

### Data analysis

The anonymised interview transcripts were initially analysed by author 1 and the generated themes were discussed and refined with the research team. Using the constant comparative method (Glaser and Strauss 1967, Silverman 1993), the researchers organised the narratives into over‐arching themes. This approach checks for the consistency of the researcher's interpretations within individual cases and across the sample group, while allowing for the specificities of each of the stories to remain. The emerging themes were also informed by considering our participants’ narratives alongside other stories that circulate about dementia (in the mainstream press for example), and identifying the extent to which our participants’ stories reflected and or resisted contemporary cultural representations of dementia and what it means to live with it. The analysis was also sensitive to the complex relationship between identity and narrative (Leiblich *et al*. 1998) helping us recognise the multiple and at times competing representations of dementia that co‐exist within individual narratives. Early iterations of the themes were shared and discussed with our study participants, the wider project team and the project advisory group to ensure resonance between our interpretations and the wider experience of those who live with and speak for people with dementia. Given the focus of our analytical work in this specific paper, the themes of the participants’ stories were also analysed through an exploration of the kinds of discourses and ideologies that underpin them (Peel 2014) as well as reflecting upon the socio‐political implications of the kinds of stories being told.

## Findings

Four key themes emerged from the data. These relate to; (i) the process of becoming a voice of or for people with dementia; (ii) biographical reinforcement and the problem of continuity; (iii) responsibilisation and the battle plan; and (iv) resistances and counter narratives.

### Becoming a voice of or for people with dementia

Narrative reconstruction following a diagnosis of illness can help to provide an antidote to the experience of feeling different or ‘abnormal’ that illness can create (Becker 1997). In the process of asking our participants about their approach to living with dementia, many described their wish to share their personal experience and tell their story of living with dementia to others. They shared a view that their story could bring benefit and enlightenment to those who hear it. For our participants, self‐narrations of living with dementia served not only to reassert their personal moral status, but also to directly challenge the value systems that create this troubling of moral status – for all those with dementia. In part, our participants stories are narratives of resistance: their accounts seek to counter pejorative and stigmatising framings of dementia through a process of re‐appropriation, taking ownership over the meanings associated with dementia that move beyond what is represented through biomedicine, the media or public perceptions.

In a similar way to Burchardt's (2016) HIV/AIDS activists, through the narrative practice of telling their stories, our participants are able to appropriate ideas about what it means to live well or ‘successfully’ with dementia. The process of becoming a voice of, or for, people with dementia therefore involves two connected narrative practices: first, people's stories act as a form of self‐presentation and identity work in which discourses of living well can be appropriated; second, these stories perform a function in the contribution they make to shaping wider perceptions of what it means to live with dementia. In the extract below, Jack, who has early onset Alzheimer's disease, describes some of the aspects of what it means to live well from his perspective:

Jack If I retain physical health then I think that has an impact upon my mental health, erm so that again helps me as well. If I'm busy then I'm living well, erm but not too busy that I'm you know overdoing it.

Researcher Yeah.

Jack Erm so I do say no to things and then I feel good I've said no to things and I write that down, in my calendar I've got all the things I'm doing but alongside I've got all the things I'm not doing you know.

Jack's descriptions are personal to the extent that they perform aspects of his identity as someone who remains socially engaged, but who is also in control of that engagement, establishing a sense of order and a ‘preservation of self’ (Beard 2004), alongside the disordering effects of the dementia. Jack's account is indicative of a discourse of dementia framed as a ‘manageable disability’ (Beard *et al*. 2009b). It is also, however, an important way in which living well or successfully with dementia is appropriated, setting out the terms of what living well might mean and how it might be accomplished while simultaneously identifying those strategies as key aspects of his own life. The appropriation of what it means to live well with dementia can also form a part of the stories told by those who live with and care for people with dementia, as this extract from Martha, the wife of Oscar who has Alzheimer's disease, illustrates:

Martha Erm, but no I think flexibility, adaptability, and finding out as much as possible about what foods to eat, erm the effect of certain medications which will have side effects. Getting good support … and the Dementia Advocacy Group which has given him a purpose.

Martha describes the kinds of personal and emotional skills required to live well with a person with dementia, as well as exercising skills to accumulate and navigate complex information related to diet and medication and finally the resourcefulness to find and obtain support for the person with dementia, in particular support that provides engagement and a sense of purpose, recognising the benefit that advocacy brings to Oscar. Martha's description is reflective of the increasingly dominant neoliberal discourse shaping health care and what it means to live well, premised on individual choice and personal responsibility (Bell and Green 2016). In providing this account, Martha is not only able to contribute to understandings of what it means to live well with dementia, but also appropriates these skills and strategies for herself, becoming a representative voice for this particular kind of ‘success’.

The more collective, social aspects of these personal stories and their purpose in shaping wider perspectives of dementia is reflected in Jack's description of speaking at a church about living well with dementia which illustrates these different but connected purposes of dementia illness narratives:

Jack I actually went to church for the first time for ages, for best part of twenty years and actually the premise behind attending was the fact that he asked me to speak to the congregation about living well with dementia. Erm because there were people in the audience, he felt, who would benefit from hearing that.

Researcher Yeah.

Jack And that was good, that was worthwhile and afterwards there was tea and coffee and so on and a number of people came up to me and commented on how helpful and interesting and uplifting the talk was but more importantly to me, on how … they were asking me questions on how their personal experience usually through a loved one, parent in most cases to be honest with you, erm was that they were … they would … their parent was dealing with dementia and by hearing me speak was helping them understand things better.

Alongside the practices of self‐presentation and identity work that shape Jack's story, we can also distinguish the nature and content of his purpose, to raise awareness and increase understanding of dementia, shaping the meanings of dementia and what it is like to live with it beyond his immediate relationships, and to extend this message to other audiences and communities.

### Biographical reinforcement, dementia and the problem of continuity

A significant thread running through the stories told by our participants about living with dementia refers to the ways in which identity and selfhood remain. The stories describe the ways in which the effects of dementia become assimilated into their everyday lives and these changes become situated in an existing identity (Beard *et al*. 2009b). This kind of story has two important implications: first, it has a performative function in helping the person with dementia to maintain a sense of continuity between past and present self; and second, it helps to construct a wider, collective voice of people with dementia that counters the claim that dementia is always and only about loss and decline and instead promotes notions of change and adaptation. This narrative reflects both the recent developments in dementia research that focus on positive approaches to living with dementia (see Wolverson *et al*. 2016) as well as to the conceptual development of personhood in understanding dementia care (Kitwood 1997, Sabat 1998). Dementia has the potential, like other forms of illness and disability, to challenge a person's life history and identity (Caddell and Clare 2010) and to create a biographical disruption (Bury 1982). However, research shows that in the responses to this threat, it is possible to identify a desire to maintain a sense of self in the face of such disruption (Caddell and Clare 2011, Harman and Clare 2006). In the case of Jack, there are attempts to make sense of these changes through a re‐interpretation of the past that reinforces components of his identity or helps build a sense of continuity and coherence, in the face of the challenges of cognitive decline. In this sense, his story is akin to Carricaburu and Pierret's (1995) concept of ‘biographical reinforcement’ as is illustrated in the following extract:

Jack To me the balance is very much different from the cognitive and the emotional where previously in my, in my working life which was let's face it the bulk of my life, erm bulk of my day I would be presenting as someone who was professional, who was erm matter of fact, who was open, who was honest, who was able to listen, absorb, remember, act upon, advise. All those things and a lot of that was largely based on, on cognitive engagement you know and let's face it, intelligence and, and, and erm abilities along the intelligence spectrum are based a lot on memory, how good a memory you've got and therefore how you can utilise what you've heard and learnt to, to do some piece of work. Now I can't do that as well as I used to be able to but what I do find is the emotional intelligence which was always there but was of secondary importance really to be honest with you, because I wanted to be, I wanted to be able to present fair professional engagement rather than an emotional engagement with somebody.

In telling this story about the shift of balance between cognitive and emotional responses, Jack is able to re‐asses his past self, as someone who did have emotional intelligence (albeit secondary to his cognitive intelligence) and is thus able to maintain a sense of coherence, making sense of now through a re‐interpretation of his past self. Jack, as Burchardt's (2016) work sets out, is becoming his own story as he speaks it, demonstrating his ability to reconceptualise and thus manage the challenges he faces as a result of his dementia. The work of talking about the past for people with dementia has increasingly been recognised as a means with which to perform important functions, including the creation and maintenance of social identities (Cheston 1996), while also helping to highlight the significant role of social contexts in shaping people's experience of their dementia (Bender and Cheston 1997). Jack's story is a good example of this function of dementia illness narratives; however it is also indicative of a resistance to the threat of stigma and social disenfranchisement (Beard and Fox 2008) that can occur as a result of the experience of dementia, by challenging commonly held beliefs about capabilities, capacity and intelligence. He identifies the significance of emotional response as being more important now, for his own being in the world, but also recognises that this form of relating to others has always formed part of his competence in social practices and interactions. Jack's appropriation of what it means to live well with dementia shifts the focus away from the cognitive decline itself and instead attributes living well to individuals’ and communities’ understanding of the multiple kinds of capacities and intelligences that enable us to relate to one another. This example therefore exemplifies the way in which Jack's personal story is able to contribute to the building of a collective illness identity through the narrative of enduring personhood.

### Responsibilisation and the battle plan

All our participants’ stories, albeit in different ways and to a varying extent, incorporate a particularly prominent public discourse that associates health with individual lifestyle choices. This association, a feature of neoliberal discourse, is part of a much wider cultural phenomenon in which individuals are increasingly seen to be responsible for managing and maintaining their own health and wellbeing as part of a project of ‘successful ageing’ (Beard *et al*. 2009a), something increasingly extended to brain health (Broer and Pickersgill 2015) and ‘staving off’ dementia. These aspects of our participants’ narratives reflect a key part of Burchardt's (2016) concept of narrative economies: that stories, in their relationships with wider public and institutional discourses, carry within them an ethical guideline for how to organise the future, so that institutional or societal demands, such as the duty to live well, become embedded in stories of personal aspirations, goals and routines (Burchardt 2016). Jane, for example, who is relatively young and has vascular dementia, describes her motivation for the community projects she is involved with that began following her diagnosis:

Jane I do it because I want people to understand it. Not for my own sanity, but you know, I look after myself but I want people to understand that young people like me can get it.

Researcher Yeah. Yeah. So that people have more, more awareness?

Jane Yes. You know, because so far years ago, it was like older people getting this memory problem and getting the Alzheimer's and getting the dementia, Things like that. So I think if you don't talk about it to, and explain to people, and get the awareness out there, just said if you don't look after yourself, this is what would happen …

Jane's story of her personal experience of living with young‐onset vascular dementia is, as she suggests, told and re‐told through her volunteering work, as a warning to others about the risks that dementia poses and to encourage people to live healthily. Jane's narrative reflects much of the current policy drive towards risk reduction in dementia, an internationally recognised commitment (World Alzheimer's Report 2014). The discourse of risk and prevention was present alongside counter‐narratives and forms of resistance, while also including multiple and diverse meanings of both the risk itself and the processes for reducing or avoiding it. For example, participants discussed their sense of responsibility and obligation to live well alongside their own mechanisms for ‘staving off’ their dementia that highlight social as well as physical practices, challenging and resisting a view of dementia that is purely somatic (Moser 2011). As we go on to show, some of our participants also explicitly challenged narratives of responsibilisation.

Oscar provided a detailed description of his ‘battle plan’ for combatting his dementia, if not to ‘beat’ it, then to alleviate its symptoms and hold off its progression:

Oscar Well, basically erm, I … I have put down in the … in my battle plan what I … what I'm doing, which keeps me going, and it's all … it's all in here, but I've got more to add now, but it's too late now. I've got what food to have, because there's certain food that you should have, which the brain wants and needs, and I've got a book full of stuff here somewhere. Er, and I adhere to that as much as I can. And I've … I'm eating vegetables and things which I haven't touched

Oscar's battle plan analogy exemplifies the power of metaphor (Sontag 1978) in ascribing meaning to the experience of illness. The battle, often situated within a wider metaphor of war with the disease constituted as the enemy, has a long standing history in accounts of living with and treating cancer (Reisfield and Wilson 2004: 4025), where there is ‘formidable chemical, biological and nuclear weaponry’. The vast arsenal required for Oscar's battle plan includes everything from listening to classical music, changes to his diet – including a daily intake of coconut oil, restricting what he eats according to his blood type, and eating vegetables that he hasn't touched before, as he describes – as well as Chinese and herbal medicines, daily exercises and finally acupressure for the brain.

The battle plan and its developing strategy is formulated through the analysis and evaluation that Oscar and his wife Martha make of various sources of evidence and information available to them. It is this that helps them to find their own approach, which incorporates some, while rejecting other, approaches to alleviating and managing the symptoms of the condition, as Martha explains:

Martha I suppose the conclusion we have come to is it's important to be holistic … because it's not just your physical wellbeing, your mental wellbeing, it's also your emotional wellbeing, and your whole lifestyle and you need to look at the whole package. Because he probably showed you Bruce Fife's coconut oil diet

Researcher Yes, yeah.

Martha Which again he launched himself into, erm, and that was, I think partially responsible for the kidney problem because he wasn't eating properly, he was having too much coconut oil, as I've subsequently found out for his … erm he follows, or we follow a diet called eat right for your blood group type. I started it when my diabetes was diagnosed and they're simple recommendations that improve your health and wellbeing.

Discourses of responsibilisation form a central part of the story of living with dementia and for Oscar and Martha this was at the core of what it means to live well with the condition. Developing their own tailored approach to strategies for ‘living well’, focused on their own individual choices and lifestyles, provided the means with which Oscar and Martha are able to gain a sense of control and order over the disordering experiences of Oscar's dementia. The narrative of responsibilisation is also nuanced by a connected and somewhat more resistant narrative in which strategies for living with dementia extend beyond the physical body; this is reflected in the inclusion of art, music, spirituality and purpose as part of Oscar's battle plan.

The circulation of stories of responsibilisation, particularly amongst those who speak both as and for those with dementia, is somewhat problematic. Such narratives can contribute to the perception that dementia is always amenable to individual modifications to lifestyle, individualising the responsibility for its development and its progression. Some have suggested that the use of the term ‘living well’ in public policy (DOH 2009) and within public health agendas more broadly, may be a further means with which to individualise both the problems and solutions associated with dementia (Peel 2014). In the case of our participants, it is important to recognise the multiple, and at times conflicting, narratives that coexist in people's stories. For Oscar and Martha, the sense of burden in their descriptions of ‘battling’ dementia is a presence throughout; however, like Jack and Fred, who we go on to discuss, there is also a sense of empowerment and resistance in their accounts, of countering the view that dementia's path is biologically fixed. Our participants were cautious in their descriptions of living well, understanding that a discourse of living well can imply that there are ways to live poorly and that this can produce a sense of blame, particularly for those living with a dementia diagnosis (Beard *et al*. 2009a).

### Resistances and counter narratives

An unease regarding the moral imperatives that are implicit in some of the rhetoric surrounding what it means to ‘live well’ with dementia or to age ‘successfully’ (Beard *et al*. 2009a), was shared by some of our participants. Jack, for example, directly challenged the concept of ‘living well’ for precisely these reasons, suggesting that ‘it should be okay to not live well’ and that the term infers a sense of responsibility or fault with the person with dementia if and when, as he puts it, they have days that are ‘quite frankly rubbish’. Jack's personal view is also beginning to be reflected in dementia research, where attempts are being made to redress the balance between recognising the suffering that comes with living with dementia alongside the possibilities for living well (Bartlett *et al*. 2017). This challenge to the living well concept mirrors some of the personal challenges of speaking as and for people living with dementia, in that efforts to represent oneself as a person living and coping with the condition can result in ‘dementia‐related fatigue’ (Bartlett 2014) akin to similar experiences of exhaustion for those with other conditions participating in health activism (see for example Earnshaw *et al*. 2016 account of people living with HIV).

A central component to Jack's story, as outlined earlier, was to shift perceptions of dementia, to understand and accept the changes that occur as a result of dementia and to incorporate these into his everyday life, maintaining a sense of identity and self‐worth. Similarly, Fred's story reflected his desire to increase awareness of dementia, not as a potential future threat to protect against, but to encourage an openness about dementia that enables people to talk about their experience and to alleviate some of the fear associated with it, a fear that can prevent people from interacting and engaging with those with dementia:

Fred It's sad sometimes when you look at it and think to yourself no one's taking no notice of the old lady in the house, you know, they're not getting … she's not coming out or anything. And you think to yourself well surely she's got neighbours that could have a chat, they don't have to come out, they can have a chat, but I think it's a stigma sometimes. You could be talking about dementia and you can see in other people's faces that they want to get away. They're under the impression that it's going to jump out of my body and go into their body.

Fred's account shows a direct awareness of the stigma associated with dementia and the ways in which stories like his about living with the condition may help to shift public perceptions and help prevent feelings of isolation and marginalisation. Illness narratives are a means with which to navigate and negotiate the problematic moral status that arises from illness; for our participants, there is an added awareness and sensitivity to the meanings attached to dementia through the stories that they tell – attending not just to their own moral status but to the value and worth attributed to all those living with dementia. This aspect of our participants stories reflects what Brown *et al*. (2004) have described as a central component of embodied health movements, which is a sense of solidarity and a collective will for a re‐appropriation of what it means to live with dementia.

In the following extract, Jack explicitly attends to the problem of circulating stories of dementia and sets out his purpose in trying to counter sad stories of loss and decline with ones that highlight how you can live with the disease:

Jack erm this was an article *(details removed)* written because somebody had committed suicide … Erm and so the [national newspaper] wanted to run a counter story which was giving a more upbeat … that it is possible to live with this wretched disease … But the point of the article is very positive so I don't have a problem with it, if it was a negative article I really would've had a problem.

Jack's account illustrates most explicitly the duality of meaning in the stories told by our participants. His personal story becomes intertwined with his sense of responsibility and awareness that stories get retold and circulate and subsequently accumulate different forms of value (Burchardt 2016). His story, and those from others like him, play an important role in either solidifying or challenging the stigma and fear associated with dementia (Devlin *et al*. 2007). Jack's reflections on his relationship with the press in this case is also an illustration of the risks Burchardt (2016) identifies as being inherent in a person's telling of their story. The more a story is told, and the more widely it circulates, the greater the potential for the story to become detached from that person's own experience.

Supporting substantial qualitative research on the experiences of people with dementia (Basting 2006, Beard 2016, de Medeiros and Doyle 2013, Harman and Clare 2006, Pearce *et al*. 2002, Sabat *et al*. 2004), our participants spoke against a singularly biomedical view of dementia. Narratives of dementia that reach beyond biomedical explanations are not restricted to those who seek to speak as representative voices of living with dementia; identifying the experiences as a ‘natural’ part of the ageing process is one example in which ‘ordinary’ people with dementia tell stories that situate their dementia outside a biomedical framework (Gillies 2010, MacRae 2008). However, in our participants’ stories, the alternatives to a biomedical view are not only presented as a personal means of understanding what is happening to them, but also constitute a strategy through which to shape wider discourses about dementia and what it means to live with it. In a similar vein to Burchardt's (2016) case of HIV activists, this aspect of our participants’ storytelling shifts the diagnosis of dementia from a medical one to a social one.

John, who is married to Julie who has Alzheimer's disease, describes his response to a psychiatrist involved in his wife's care:

John I think your only solution for dementia is love. None of the … none of the pharmaceutical products work at all. I mean I said to the psychiatrist, err, you know, slightly aggressively, you don't do anything, you can't do anything and she had a student in and I told the student do something else. She's not bad, it's not that bad, but I … I said to her in the hall you … you can't do anything for these people. She made a very profound reply. She said we try to help the families.

John's story reflects a particular kind of expectation placed upon clinicians who care for people with dementia. John's frustration over what this psychiatrist is and is not able to do is perhaps indicative of an over‐emphasis on pharmacological interventions for the treatment and cure of dementia, something mirrored in the historical allocation of research funding in the field (Cohen‐Mansfield and Mintzer 2005). Such an emphasis may threaten recognition of the benefits of social and psychological interventions and support for people living with dementia. In pointing out what John sees as the inability of clinicians to provide this particular kind of help, John accounts for what can be done to care for people with dementia as something distinct from professional care as he sees it. In particular, he advocates love, which he sees as being separate to the offerings of professionals and instead situates this support within the realm of personal relationships and the support networks of family and friends.

This challenge to a purely biomedical model for understanding what dementia is and how to live with it highlights a more nuanced account that draws on embodied, relational and – in the case of the extract above – emotional and spiritual understandings of the effects of dementia and how to live with its challenges. According to our participants, and building upon one of the founding premises of the IDEAL study, living with dementia attends more to the social aspects of life and far less to biological functions, as this extract, also from John, describes:

John So we have somebody coming in twice a week, 2 hours a week now. And then we're looking at a care home … a care … a care activity centre for 1 day a week. And then we go to Tai Chi and then Julie's studying, um, piano lessons. So I mean, for example, now she would get quite upset that she wasn't doing something now. She'd get confused. But she's now starting to watch much much more good straightforward television. Not films, she can't cope with more than an hour even. Half an hour's probably better and she can't follow a plot very well, err, but if … but I think you need to have activities, whatever the level of the person.

Focusing on activities, the social engagement and purpose that they offer, is indicative of the kinds of counter‐narratives our participants proposed in relation to living with dementia. Accounts that enable and support people to maintain a sense of identity and self‐worth while also experiencing loss of memory or other symptoms of dementia (Latimer 2013). This continued activity and sense of purpose was very much echoed in the story told by Julie herself, who also described helping those within her church on financial matters (after retiring from her accountancy career):

Julie And, em, er, and there was plenty of things I could do at church too, you know, because … they, they want certain things doing. And, and, you don't lose all the, the information that you have … And so somebody comes in with a question and you can answer it, you can answer it. Em, so.

Julie's account of her engagement with her church contributes to a picture of continuity in the lives of people living with dementia, in which purpose and activity can remain, even alongside the challenges that the condition can create.

## Discussion

The voices of people with dementia are increasingly being recognised in both policy and research. Our purpose in this article has been to reflect on one group of people who seek to contribute to this growing social movement and to consider their stories – not only as personal accounts that carry experiential authenticity – but also as narrative resources, available as currency in a narrative economy of dementia. Although our interviews were not directly focused on our participants’ roles as spokespeople for dementia, the participants all spoke about sharing their story as an important part of living well with dementia and finding meaning and purpose in contributing to a greater understanding of dementia amongst researchers, policymakers, practitioners and in the wider communities in which they live. Such accounts are unsurprising, given that our participants were already keenly involved in such activities. Our participants’ stories provide an important insight into the duality of dementia illness narratives, highlighting both the personal nature of their contents as well as their wider social, cultural and political framing. This duality is a necessary component of what gives these stories their value – they must be simultaneously distinct, embodied and ‘authentic’ while also operating on a communal level, reflecting a shared experience of living with dementia (Mazanderani *et al*. 2013). Part of our project in this paper has been to develop a process of linking up the personal and the political in the narrative accounts of those who seek to speak for what it is like to live with dementia, contributing to what Baldwin (2008) describes as a form of narrative citizenship. As a means of contributing to this project, we show how an important first stage of making this connection is to appropriate the very strategies and attributes that are described as what it means to live well with dementia.

The moral quest in illness narratives (Hyden and Orulv 2009) is identified through our participants’ stories, particularly in practices of biographical reinforcement whereby aspects of the condition that could threaten moral status are re‐interpreted. While recognising this identity work, we also show how this attention to moral status incorporates strategies for producing collective representations of dementia and even challenges the values that underpin this threat to people's value and moral status. In identifying the dramaturgy of responsibilisation we have shown the connections with, and circulation of, more dominant narratives of dementia and their reproduction. The presence of this dramaturgy is partly a reflection of the actively engaged advocates who made up our participant group, who are perhaps more likely to be aware of and appropriate discourses of reponsibilisation. Although identifying the potentially problematic and stigmatising effects of this narrative thread, we also situate this story within the multiple and layered stories from our participants, including accompanying counter‐narratives and resistances – illustrating that stories are never entirely linear or singular (Leiblich *et al*. 1998).

Finally, we attend to the counter‐narratives set out by our participants, engaging in challenges to biomedicine and pharmacology as well as providing alternatives to a narrative of inevitable loss and decline. Although these stories are highly personal and therefore varied in their detail, they are organised through similar kinds of dramaturgies (Beard 2004, Burchardt 2016) that tell stories of a continued sense of identity and personhood beyond that of cognitive decline, displacing the metaphor of a living death with stories of living *with* dementia, adapting to difficulties in ways that help maintain a sense of self (Beard *et al*. *20*09). This dramaturgy is able to connect the personal nature of the stories to broader concerns that contribute to a potentially distinct collective illness identity that is specific to dementia. Other chronic illnesses carry the potential for a loss of self (Charmaz 1983) that requires narrative reconstruction. However, the focus on retained selfhood in the face of cognitive decline is unique to dementia. Dementia illness narratives, as told by our participants, seek to combat the stigma associated with the condition itself – through its representation as a gradual loss of self ‐ and increased dependence, which can negatively impact upon a person's social and moral value (Grenier *et al*. 2017).

If we are to understand these narratives as commodities (building on Burchardt 2016), we must question the implications of their accumulation and circulation to ascertain the kinds of value – both symbolic and material – they produce through a recognition of particular aspects of the dementia experience. Of course, our participants’ stories reflect the experiences of those who are highly engaged, aware of and responsive to the kinds of images and representations that circulate around dementia, and, perhaps more importantly, feel a responsibility to challenge and redress discourses that are stigmatising. In the quest to give voice to those living with dementia, we must equally consider which voices remain unheard and what kinds of stories may be absent as a result. As Burchardt's (2016) work illustrates, some stories may accumulate more value than others, particularly in the context of campaigning, fundraising and the quest to ascertain political and social recognition. Perhaps the voice of the frail older person with dementia, who is more likely to incorporate the experience of dementia into a broader story of ageing (see for example the stories represented by Sullivan and Beard 2014), produces less value in the narrative economy of dementia? How might people with these different kinds of stories relate to the narratives of those who are increasingly called upon to speak for them? These are important questions in seeking to understand the relationships between the experiences of living with dementia, dementia activism and institutions of dementia.

To develop this empirical and conceptual work, it is important to examine which stories gain traction and which are constrained in their circulation through the various institutions of dementia such as research, medicine, clinical practice and care, dementia policy and in wider public discourses of the condition. We suggest, particularly drawing on the framework of narrative economies, that this field of research requires further study and attention. Given the proliferation of dementia illness narratives, it is important to consider the ways in which they are or are not taken up, the processes through which they are made to represent dementia and what kinds of value they produce. This is particularly salient given Burchardt's (2016) warning of a potential disconnect between a story that accrues value through its circulation, and the storyteller's lived experience. Our work has undertaken a first step in exploring the wider discourses and conceptions of dementia that are being produced and identified through the narratives of some of those who represent what it means to live with dementia. The next stage requires some ethnographic exploration, to identify which narratives gain traction, are re‐told and accumulate value, which stories are made absent and what are the social and political implications of these narratives for those living with dementia.

Our participants’ stories reflect ongoing tensions between a hegemony of biomedical interpretations of dementia (Beard *et al*. 2009a) and the compelling nature of its depiction as tragedy and loss on the one hand, and the socially shaped nature of its lived experience on other, whereby the meanings associated with dementia are shaped by people's relationships, their environments and the wider communities in which they live. What these stories capture are also the ways in which illness narratives are rarely coherent and linear but are instead multi‐layered and at times contradictory. The nature of our group of participants meant that although there was some mirroring of wider cultural preoccupations with responsibilisation and the duty to stave off dementia, there was a stronger sense of resistance and re‐appropriation in their stories through which they sought to take ownership over what it means to live well with dementia.

## Note

1 FADE Campaign, Alzheimer's Society, http://www.fadetoblank.org/#/?part=cause-for-concern
